# 576. Comparison of Three Blood Culture Testing Models at a Tertiary Care Hospital: Does Laboratory Centralization Work?

**DOI:** 10.1093/ofid/ofad500.645

**Published:** 2023-11-27

**Authors:** Aya Haghamad, Vincent Streva, Stefan Juretschko, Jamie Lemon

**Affiliations:** Northwell Health laboratories, Little Neck, New York; Northwell Health Laboratories, Queens, NY; Northwell Health laboratories, Little Neck, New York; Northwell Health Laboratories, Queens, NY

## Abstract

**Background:**

Centralization of laboratory testing has become increasingly common in US hospital systems. One criticism of centralization is increased test turnaround time leading to potential delays in care.

**Methods:**

In this study, we analyzed three microbiology laboratory testing models for blood cultures at a tertiary care hospital. These models were: 1) full workup in a hospital-based microbiology laboratory; 2) incubation, Gram stain (GS), and molecular blood culture identification (BCID) in a hospital-based microbiology laboratory followed by subculture identification and susceptibility testing (AST) at a centralized core laboratory; and 3) full workup in the centralized microbiology laboratory. Blood culture samples were transported 33 miles from the hospital to the core laboratory. We evaluated these three models for time from collection to positivity, time from collection to GS result, time from collection to BCID result, time from collection to organism identification, time from collection to AST, and patient length of hospital stay.

**Results:**

We observed no statistical difference in time to blood culture positivity, time to GS result, or time to BCID result. There were shifts in the median time to AST between Model 1 (53.6h) and Model 2 (73.9h) and Model 2 and Model 3 (63.8h), but these were not statistically significant. Of note, time to AST and time to final result in Model 3 were more uniform, with fewer outliers than in the other two models. This is likely a result of the greater degree of laboratory automation in place at the core laboratory and reduced time spent in transit before culture workup. Finally, there was no difference in length of hospital stay between the hospital and decentralized models, though the hybrid approach (Model 2) resulted in increased length of hospital stay compared to either of the other models.

**Conclusion:**

Our results suggest centralization of blood culture testing does not significantly impact timely reporting of laboratory results and has no impact on length of patient hospital stay. Interestingly, these data demonstrate potential delays when blood culture workups are split between hospital and centralized laboratories. Additional work is needed to fully understand the role of centralization of laboratory testing on treatment decisions and patient outcomes.

**Disclosures:**

**All Authors**: No reported disclosures

